# Effects of Age on Long Term Memory for Degraded Speech

**DOI:** 10.3389/fnhum.2016.00473

**Published:** 2016-09-21

**Authors:** Christiane M. Thiel, Jale Özyurt, Waldo Nogueira, Sebastian Puschmann

**Affiliations:** ^1^Biological Psychology Lab, Cluster of Excellence “Hearing4all”, Department of Psychology, European Medical School, Carl von Ossietzky Universität OldenburgOldenburg, Germany; ^2^Research Center Neurosensory Science, Carl von Ossietzky Universität OldenburgOldenburg, Germany; ^3^Cluster of Excellence “Hearing4all”, Department of Otolaryngology, Medical University HannoverHannover, Germany

**Keywords:** vocoded speech, long term memory, working memory, verbal IQ, age

## Abstract

Prior research suggests that acoustical degradation impacts encoding of items into memory, especially in elderly subjects. We here aimed to investigate whether acoustically degraded items that are initially encoded into memory are more prone to forgetting as a function of age. Young and old participants were tested with a vocoded and unvocoded serial list learning task involving immediate and delayed free recall. We found that degraded auditory input increased forgetting of previously encoded items, especially in older participants. We further found that working memory capacity predicted forgetting of degraded information in young participants. In old participants, verbal IQ was the most important predictor for forgetting acoustically degraded information. Our data provide evidence that acoustically degraded information, even if encoded, is especially vulnerable to forgetting in old age.

## Introduction

The richness of acoustic signals is an important factor that contributes to speech intelligibility. Many everyday situations are however characterized by factors that impact acoustic richness such as competing speakers, increased background noise or subject-specific factors like age-associated hearing loss, even if compensated with hearing aids. Despite this reduction in acoustic richness, listeners are usually able to extract information from degraded speech signals (Davis et al., 2005). Speech comprehension is however slower and less efficient in these situations (e.g., Wagner et al., 2016).

Several lines of evidence further suggest that acoustic degradation may impact memory because degradation draws on resources that are no longer available for encoding of items into memory. For example, it has been shown that immediate recall and associative memory decline in young adults presented with experimentally degraded stimuli. The performance decline mimics the performance of older adults with age-related hearing loss—even if the stimuli are presented with enough clarity to be understood (McCoy et al., 2005; Surprenant, 2007; Piquado et al., 2010; Heinrich and Schneider, 2011; Naveh-Benjamin and Kilb, 2014). The finding of reduced immediate memory for speech stimuli presented in noise or in temporal proximity to noise, has already been reported by Rabbitt (1968), who suggested that the effect depended on the increased effort necessary for stimulus recognition that prevents it’s adequate encoding. Tun et al. (2009) tested this suggestion of increased effort in a dual task condition where listeners with hearing loss were presented with spoken word lists. As expected, poor hearing status increased dual task costs on immediate recall, an effect that was exacerbated in elderly subjects. Several recent neuroimaging studies provide evidence for the neural consequences of degraded auditory input. They compellingly demonstrate that even a mild to moderate hearing impairment leads to an impoverished representation of auditory input in speech processing regions such as the superior temporal gyri and reduced structural integrity of primary auditory cortex (Peelle et al., 2011). Moreover, studies in young and elderly volunteers with age-appropriate hearing provide further evidence that the processing of degraded auditory input co-occurs with increased activation in a cingula-opercular network and this compensatory activation in non-speech areas is related to increased speech recognition (Wild et al., 2012; Erb et al., 2013; Vaden et al., 2016).

Aging impacts both, auditory processing, especially in challenging situations, and cognitive function such as working and long term memory. For example, hearing-impaired elderly subjects show stronger impairments in understanding linguistically complex sentences than younger subjects with the same hearing impairment (Wingfield et al., 2006). Additional evidence supports the interaction between the loss of acoustic detail and verbal memory because memory in older adults was found to be stronger affected by acoustic degradation (Heinrich and Schneider, 2011). The relation between cognitive and sensory decline in old age has been investigated extensively (e.g., Lindenberger and Baltes, 1994; Baltes and Lindenberger, 1997) and it was shown that hearing loss is negatively related to episodic and semantic long term memory even in elderly subjects who compensated their hearing loss with hearing aids (Rönnberg et al., 2011). Note, that this effect is not solely due to a sensory degradation, which may not be completely compensated by the hearing aid, since a negative correlation was also found between visually tested prospective memory and hearing loss in a large cross sectional epidemiological study (Rönnberg et al., 2014).

Working memory is crucial for simultaneously processing and storing information and a wealth of experimental evidence relates working memory capacity to speech intelligibility, particularly in adverse listening conditions (for review see Akeroyd, 2008; Rönnberg et al., 2013; Rudner and Lunner, 2014). That working memory may also play a causal role in long term memory decline was recently shown by Hara and Naveh-Benjamin (2015), who manipulated working memory in young healthy volunteers and were able to reproduce the associative memory deficit observed in old age. We here aimed to investigate to what extent degraded auditory input impacts on consolidation of information into long term memory. In addition, we aimed to explore in the current dataset, how these effects are related to working memory capacity. While the research reviewed above suggests that degraded auditory information impacts initial encoding, it is not known whether degraded information, that is initially encoded, undergoes consolidation to the same extent as non-degraded information or whether degraded information is more fragile and prone to forgetting.

We choose for an experimental degradation of auditory input rather than the natural degradation present in age-related hearing loss since the comparison of hearing impaired subjects with a control population is often confounded by age given that hearing impairments get more prevalent with increasing age. We presented young and old participants with a vocoded and unvocoded version of a standardized verbal list learning task that had to be recalled immediately on successive trials as well as after a 30 min delay. Forgetting was gauged by comparing immediate and delayed recall. We hypothesized that vocoded information should be more vulnerable to forgetting and that the effect should increase in old age. Further, we hypothesized that a higher working memory capacity may counteract forgetting of vocoded information given prior evidence that individual differences in forgetting in elderly volunteers are strongly related to working memory capacity and processing speed (Zimprich and Kurtz, 2013).

## Materials and Methods

### Subjects

Twenty-one younger (18–34; mean: 23.9; 13 female) and 20 older (55–74; mean: 65.15; 13 female) adults participated in the study. All participants were right-handed, native speakers of German and had an above average verbal IQ as tested with a multiple choice word test that requires participants to select the correct word among five distractor non-words and hence tests for vocabulary size (WST, Schmidt and Metzler, 1992). All subjects had age appropriate hearing which was defined as less than 20 dB HL between 125 Hz and 8 kHz in young participants and less than 25 dB HL for individual frequencies below 3 kHz as well as less than 20 dB HL combined over the frequencies of 500 Hz, 1 kHz, 2 kHz and 4 kHz in old participants. Participants with any significant neurological or psychiatric conditions were excluded. Ethics approval was obtained from the local ethics committee. The study was conducted in accordance with the Declaration of Helsinki, and all procedures were carried out with the adequate understanding and written informed consent of all participants. Four subjects in the older group and one subject in the younger group had to be excluded from the analysis for the following reasons: two subjects aborted the working memory task, one subject had a working memory score of 0, one subject did not succeed to learn the vocoded speech (vocoded speech understanding of 0) and another subject showed no learning of the vocoded word list in the verbal learning and memory test (immediate recall score of 0).

### Procedure and Tests

Participants took part in two testing sessions of 90 min each on two subsequent days. All testing was conducted in a double walled sound attenuating booth. Auditory stimuli were presented binaurally via Sennheiser HD 250 linear II headphones at 76.5 ± 0.87 dB.

### Verbal Learning and Memory Test (VLMT)

**Figure 1:**
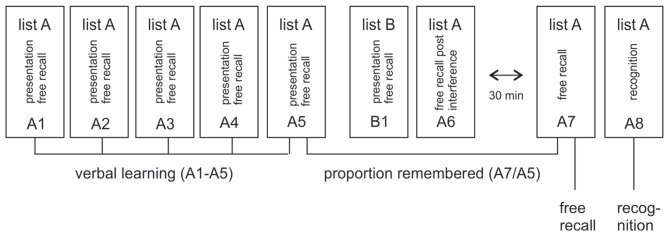
**Illustration of verbal learning and memory test (VLMT).** The test consisted of five repeated auditory presentations of word list A (A1–A5). After each presentation the subject had to freely recall as many words as possible. This was followed by the presentation and immediate free recall of a second, interfering list (list B, B1) and the subsequent requirement to recall list A again after this interference (A6). Memory was assessed after a 30 min break with free recall of list A (A7) and a following recognition test (A8). The main measure of interest in this article is the proportion of remembered items after the 30 min break in relation to the last immediate free recall presentation (A7/A5). In addition, verbal learning (i.e., the proportion of all words immediately remembered in lists A1–A5), free recall (i.e., the proportion of items freely recalled after the delay in A7) and recognition (i.e., number of words recognized from a larger list of verbally presented words after the delay in A8) were reported.

Learning and memory was assessed with a standardized multitrial learning task (Lux et al., 1999) that consisted of five repeated auditory presentations of a 15-word list (list A) that had to be recalled immediately after each presentation (A1–A5), see Figure 1. This was followed by the presentation and immediate free recall of a second 15-word list (list B, B1) and the subsequent requirement to recall list A again (A6) after this interference. Memory was assessed after a 30 min break with free recall of list A (A7) and a following recognition test (A8). The recognition test consisted of 50 verbally presented words, the 15 target words from list A, 15 distractor words from list B and 20 new words. The subjects’ task was to indicate with a yes/no response whether a word was from list A. To study the effects of degraded auditory input on verbal learning and memory, we used two parallel versions of the VLMT. Both versions were spoken by a female speaker, recorded and vocoded as detailed below. During testing, participants listened to the vocoded and unvocoded audiofiles via headphones. Verbal responses were recorded and scored by the experimenter. In the vocoded condition, only words that matched the correct word were scored as correct. Data analysis focused on memory loss after the delay, indexed by the proportion of freely recalled items after the 30 min break in relation to the last immediate free recall presentation (A7/A5) and labeled as VLMT proportion remembered. This measure was chosen as primary outcome rather than the immediate or delayed recall *per se*, because it is not confounded by the number of words understood by each individual participant. The measure therefore enables a comparison between the vocoded and unvocoded condition even with reduced performance under vocoded speech conditions. Immediate recall (i.e., the proportion of words recalled over all lists A1–A5, labeled as VLMT verbal learning), delayed recall (proportion of words remembered after the delay A7, labeled as VLMT free recall) and delayed recognition (proportion of words recognized from the list of words after the delay A8, labeled as VLMT recognition) were reported for completeness and comparison with prior studies. Note that the performance in B1 and A6, which are part of this standardized test, can be used to measure memory after interference. Since this was however not the focus of the present study this data were not analyzed.

### Vocoding and Training

Acoustical degradation was produced by using a noise-vocoding technique that preserves the temporal information of the speech envelope but reduces the spectral information. The noise-vocoder simulated the typical processing performed by a cochlear implant and the spread of excitation that may occur in the electrically stimulated cochlea (Nogueira et al., 2016). Each token was digitally sampled at 16 kHz. A 128 point short time fast Fourier transform (FFT) was computed with a 75% overlap. Next, the FFT bins were grouped into 10 non-overlapping, logarithmically spaced bands. The envelope of each band was computed taking the square root of the total energy in the band. The output of each band was used to modulate a noise band. The noise band was generated similarly synthesized in the frequency domain (Litvak et al., 2007). The center frequency of the noise band was identical to the center frequency of the corresponding frequency band. Further, the noise band was configured to decay at a rate of 3.5 dB/octave to simulate the effect of spread of excitation. The specific parameters of the vocoder were chosen after piloting to produce speech that was difficult to understand initially but allowed learning of this degraded input with short training, both in young and older volunteers. Subjects were trained on vocoded sentences prior to the experiment. The training consisted of a short presentation of eight vocoded seven word sentences taken from Uslar et al. (2013) which were repeated back to the participant in clear speech. This was followed by the presentation of further 60 seven word sentences which participants were required to repeat back aloud. The number of correctly repeated words in these 60 sentences were taken as an index of understanding acoustically degraded speech (vocoded speech understanding). The number of correctly repeated keywords was scored per sentence. Correct words with a wrong case or grammatical suffix were also scored as correct. Vocoded sentence learning was performed immediately before the presentation of the vocoded version of the VLMT.

### Operation Span Task

Working memory capacity was assessed with the operation span (OSPAN) task according to Unsworth et al. (2005). In this task, participants are required to solve a set of simple arithmetic operations while trying to remember a letter after each operation for later recall. The set size after which a recall is requested varies randomly from three to seven. For recall, participants were presented visually with a 12 letter matrix and requested to choose the letters in the same order in which they had appeared by mouse click. Working memory capacity was gauged by adding the number of items recalled in correct order in all memory trials (OSPAN score). On both days, participants performed additional neuropsychological tests which are not reported here (trail making test, Stroop test, text reception threshold test and the lexical decision task).

### Statistics

Statistical analysis was performed with SPSS23 if not otherwise indicated. For each performance measure means and standard errors of means were computed across subjects. Normality of all measures entering into the analyses was tested with Kolmogorov-Smirnov tests in each group. None of the measures deviated from a normal distribution. Performance differences between young and old subjects in the tests used were assessed by means of independent samples *t*-tests using Bonferroni adjusted alpha levels (*p*-value*11). Verbal learning over the five presentation trials was tested in the unvocoded and vocoded condition with an analysis of variance (ANOVA) for repeated measures with the within subject factors trial (A1–A5) and degradation (vocoded/unvocoded) and the between subject factor age (young/old). To assess the effects of acoustically degraded sensory input and age on memory loss, indexed by the proportion of remembered items (A7/A5), we performed an ANOVA for repeated measures with the within subject factor degradation (vocoded/unvocoded) and the between subject factor age (young/old). Vocoded speech understanding and verbal IQ were entered as covariates. To further test the hypothesis that a high working memory capacity prevents memory loss of sensory degraded information and that this may be especially relevant in older volunteers, we performed two stepwise multiple linear regression analyses in young and old volunteers (inclusion *p* < 0.05, exclusion *p* > 0.1). Memory loss was again indexed by the proportion of remembered items. To focus on specific effects of memory loss of sensory degraded information this measure was calculated as a proportion of performance in the non-degraded condition [i.e., (A7_voc_/A5_voc_)/(A7_unvoc_/A5_unvoc_)], labeled VLMT proportion remembered (vocoded/unvocoded). This variable was entered as dependent variable into the model. The predictors entering the model were the OSPAN score as our main predictor of interest and vocoded speech understanding and verbal IQ.

## Results

Table 1 displays relevant measures of mean test performance in young and old participants and significant differences between age groups after Bonferroni correction. As expected older adults showed significantly reduced performance in several aspects of verbal learning and memory and working memory. They also showed lower rates of vocoded speech understanding, even though this measure was not significant after correction for multiple comparisons and individual data showed a large overlap of performance (young volunteers: minimum of 10% vocoded speech understanding, maximum of 99%; old volunteers: minimum of 10%, maximum of 90%). Further, older adults had higher verbal IQ estimates.

**Table 1:** **Test performance (mean and SEM) and group differences (*p*-value of independent samples *t*-test)**.

	Young	Old	*p*Bonf
WST: IQ estimate	104 ± 1.3	113 ± 2.03	0.011*
Vocoded speech understanding	0.62 ± 0.06	0.40 ± 0.06	0.099
Working memory: OSPAN	60.2 ± 2.0	45.8 ± 3.6	0.022*
VLMT proportion verbal learning (mean A1–A5)
unvocoded	0.86 ± 0.02	0.77 ± 0.03	0.154
vocoded	0.48 ± 0.04	0.28 ± 0.04	0.011*
VLMT proportion verbal learning last trial (A5)
unvocoded	0.96 ± 0.01	0.85 ± 0.03	0.033*
vocoded	0.56 ± 0.04	0.32 ± 0.05	0.011*
VLMT proportion free recall (A7)
unvocoded	0.96 ± 0.02	0.75 ± 0.04	<0.001*
vocoded	0.52 ± 0.05	0.23 ± 0.04	0.001*
VLMT proportion recognition (A8)
unvocoded	0.98 ± 0.008	0.91 ± 0.02	0.187
vocoded	0.81 ± 0.04	0.62 ± 0.04	0.066

pBonf = Bonferroni corrected p values (p-value*11). Significant group differences are indicated by a star.

**Figure 2:**
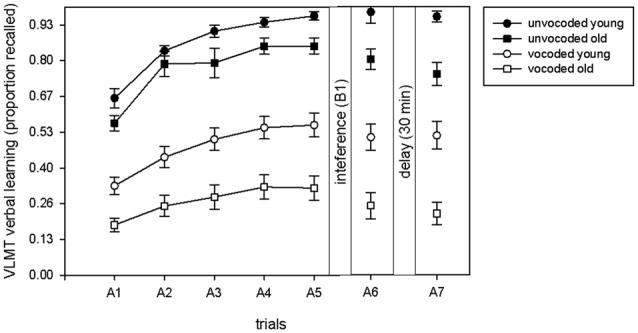
**Proportion of recalled items in the learning phase (A1–A5), after interference (A6) and after the delay (A7) in the VLMT as a function of degradation and age.** Learning was evident to a similar extent under all conditions and in both age groups.

In order to compare our dataset to the previously reported data (McCoy et al., 2005; Surprenant, 2007; Piquado et al., 2010; Heinrich and Schneider, 2011; Naveh-Benjamin and Kilb, 2014) on immediate recall of degraded information, we illustrate the proportion of immediately recalled items in the five learning trials as a function of degradation and age (Figure 2). A significant increase in the proportion of recalled items over the five learning trials occurred across all conditions (ANOVA main effect of trial *F*_(1,34)_ = 234.4, *p* < 0.001). Even though old volunteers recalled less items in both conditions (ANOVA main effect of age *F*_(1,34)_ = 18.61 *p* < 0.001), there was no significant difference in learning between young and old volunteers in general (trial by age interaction *F*_(2.5,85.1)_ = 1.71, *p* = 0.13) or in learning in young and old volunteers between the unvocoded and vocoded condition (trial by age by degradation interaction (*F*_(4,136)_ = 1.14 *p* = 0.46). There was however a trend for a significant difference between old and young volunteers with respect to overall performance in the vocoded vs. unvocoded condition (age by degradation interaction, *F*_(1,34)_ = 3.05, *p* = 0.09). Note that this effect was similar, if only the last learning trial, A5, was taken into account (age by degradation interaction, *p* = 0.083).

**Figure 3:**
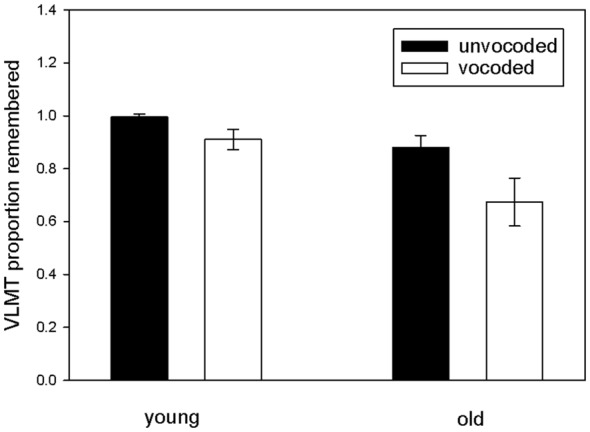
**Proportion of remembered items in the VLMT as a function of degradation and age.** Proportion of remembered items was indexed by the ratio of recalled items after the 30 min break in relation to the last immediate free recall presentation (A7/A5). Note the lower rates of memory for vocoded items in old participants.

To investigate our main hypothesis, that sensory degradation impacts consolidation of auditory information into long term memory, we focused on the proportion of remembered items after the delay as a function of degradation and age and performed a repeated measures ANOVA on this data with vocoded speech understanding and verbal IQ as covariates (see Figure 3). The results revealed significantly reduced long term memory for vocoded items (ANOVA main effect of degradation *F*_(1,32)_ = 7.3 *p* = 0.011), significantly reduced long term memory for vocoded and unvocoded information in old as compared to young volunteers (ANOVA main effect of age *F*_(1,32)_ = 8.65 *p* = 0.006) and, most importantly, a degradation by age interaction (*F*_(1,32)_ = 6.57 *p* = 0.015) which confirms the hypothesized stronger forgetting of vocoded items in the elderly. There was also a degradation by verbal IQ interaction (*F*_(1,32)_ = 6.15 *p* = 0.019) which indicates stronger forgetting of vocoded items in subjects with lower verbal IQ. To rule out that the results are confounded by a ceiling effect in young volunteers in the unvocoded condition, we performed a *post hoc* analysis where we excluded those volunteers performing at ceiling (*n* = 14). Even with this small sample size effects were similar (significant main effect of degradation *F*_(1,18)_ = 7.36 *p* = 0.014), tendency for significant main effect of age *F*_(1,18)_ = 3.43 *p* = 0.081), and a tendency for degradation by age interaction (*F*_(1,18)_ = 3.65 *p* = 0.072).

**Figure 4:**
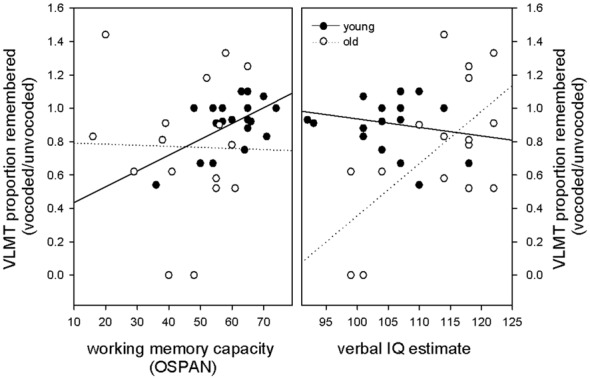
**Relationship between the proportion of remembered vocoded items (relative to unvocoded items), working memory capacity and verbal IQ.** Note that in young participants, higher working memory capacity but not verbal IQ predicted better memory for degraded information, whereas the opposite pattern was found in old participants where verbal IQ was the only predictor.

Given that working memory capacity is related to forgetting and speech understanding in adverse listening conditions, we hypothesized that a high working memory capacity should counteract forgetting of vocoded items. Again, we hypothesized that this effect may be specifically evident in elderly volunteers that have comprised working memory. To focus on effects of memory loss that are specific to degraded information this measure was calculated as a proportion of performance of the non-degraded condition [VLMT proportion remembered (vocoded/unvocoded)]. To test the hypothesis, we performed in each age group a stepwise multiple linear regression with OSPAN score, vocoded speech understanding and verbal IQ as predictor variables and VLMT proportion remembered (vocoded/unvocoded) as dependent variable. We expected that the OSPAN score would appear as significant predictor for memory loss, especially in the elderly. Our results revealed that working memory was the only predictor in the group of young subjects and explained a significant amount of the variance in the proportion of remembered (vocoded/unvocoded) items (*F*_(1,18)_ = 8.44 *p* = 0.009 *R*^2^ = 0.319, *R*^2adjusted^ = 0.281, beta = 0.565, *T* = 2.90 *p* = 0.009). All other predictors were not significant with verbal IQ at *p* = 0.655 and vocoded speech understanding at *p* = 0.602. With respect to the group of old subjects, our results revealed no evidence for working memory as a significant predictor of forgetting degraded information. The only significant predictor in the group of old subjects, that explained a significant amount of the variance in the proportion of remembered (vocoded/unvocoded) items was verbal IQ (*F*_(1,14)_ = 8.45 *p* = 0.011 *R*^2^ = 0.376, *R*^2adjusted^ = 0.332, beta = 0.614, *T* = 2.91 *p* = 0.011). All other predictors were not significant with working memory at *p* = 0.279 and vocoded speech understanding at *p* = 0.447. In other words, in contrast to our expectations, working memory capacity did only predict forgetting of degraded auditory information in young, but not old volunteers. Figure 4 illustrates these findings in showing that in young participants, higher working memory capacity predicted better memory for degraded information, whereas no such relationship was found in old volunteers. Regression slopes were significantly different between groups (*t*_(32)_ = 2.1 *p* = 0.04). In contrast, in old participants verbal IQ was the only predictor for forgetting degraded information with higher verbal IQ predicting better memory for degraded information. No such relationship was found in young volunteers. Regression slopes were significantly different between groups (*t*_(32)_ = 3.13, *p* = 0.004). The robustness of the described effects against outliers was probed using an additional robust regression analysis (robustfit, MATLAB Statistics Toolbox) with a logistic weighting function (tuning constant = 1.205). Matching the findings obtained with the ordinary least-square regression, the analysis showed a significant relationship between forgetting of degraded information and working memory capacity in young but not old volunteers (young: *R*^2^ = 0.270, *p* = 0.019, old: *R*^2^ = 0.00006, *p* = 0.977) and a significant relationship between forgetting of degraded information and verbal IQ in old but not young volunteers (old: *R*^2^ = 0.311, *p* = 0.025, young: *R*^2^ = 0.00005, *p* = 0.975).

## Discussion

The present article aimed at investigating the relationship between consolidation of acoustically degraded items into long term memory and age. Our results suggest that degraded auditory input increases forgetting of previously encoded items, especially in older participants. An additional exploratory analysis examined how individual working memory capacity is related to forgetting of degraded auditory input. This analysis, which needs to be replicated in a larger dataset, suggests that working memory predicts forgetting only in young participants. In old participants, verbal IQ was the most important predictor for forgetting acoustically degraded information.

Overall, the performance data confirm the previously reported superior performance in younger as compared to older adults in all cognitive domains tested. Better adaptation of young volunteers to degraded auditory input was previously found with respect to time compressed and noise-vocoded speech (Peelle and Wingfield, 2005; Sheldon et al., 2008; Neger et al., 2014). Therefore, different vocoding schemes that resulted in less degradation in elderly volunteers were used in some prior publications (Neger et al., 2014). Given that piloting with our vocoded sentences revealed however a strong overlap of performance in young and older volunteers, and given that the measure of long term memory used here did not depend on how many items were understood in the first place, we refrained from using different levels of degradation in young and older participants. Additionally, vocoded speech understanding was entered as covariate into our analyses, so that performance differences with respect to vocoded speech understanding are unlikely to have confounded our findings. The second covariate entered into the analyses was verbal IQ since higher vocabulary scores are common in older adults (Peelle and Wingfield, 2005; Sheldon et al., 2008; Neger et al., 2014) and were also evident in our sample of participants.

The increased amount of forgetting of degraded verbal information extends prior findings, focusing on initial encoding of naturally or experimentally degraded auditory information (McCoy et al., 2005; Surprenant, 2007; Piquado et al., 2010; Heinrich and Schneider, 2011; Naveh-Benjamin and Kilb, 2014). That this amount of forgetting is stronger in older volunteers is in line with previous findings (Tun et al., 2009; Heinrich and Schneider, 2011), our effects however relate to the consolidation and later retrieval of degraded verbal information. Note that our own data did only show a trend for a significantly different decline of immediate recall under vocoded conditions in young and older participants. We therefore conclude from our own data that degraded auditory information may be encoded to a similar extent in younger and older participants, the memory trace of vocoded items is however more fragile with increasing age, which is reflected by the significantly increased rate of forgetting in elderly subjects. Our additional analysis, where we tried to equate performance across young and old volunteers as far as possible suggests that this increased forgetting in elderly as compared to young subjects is at least not driven to a strong extent by a ceiling effect in young volunteers. Nevertheless, age-related differences in memory performance may always bias results if groups are not large enough to enable perfect *post hoc* matching of memory performance across age groups.

The increased fragility of the memory trace may be explained by limited resources available for proper encoding which may impact to a larger extent on delayed recall which is more taxing. Comparable results were reported by Naveh-Benjamin and Kilb (2014), who showed that young subjects tested under degraded conditions did not show any impairments in immediate recall of words *per se*, however, the more taxing memory component, immediate associative memory of word pairs, was impaired. Since vocoded speech simulates the auditory signal of a cochlear implant, our findings, that suggest that a loss of acoustical richness may not significantly impact immediate simple word recall, may seem at odds with recent data in cochlear implant users. Pisoni et al. (2016) studied prelingually deaf long term cochlear implant users and provide evidence for reduced immediate word recall in a list learning task. The authors suggest that early encoding and storage of information is crucial for speech and language processing and that this process is comprised in subjects with cochlear implants. Unfortunately, memory loss after the delay was not reported in that study, so that delayed recall cannot be compared to our data. Nevertheless, the authors described that cochlear implant users were more likely to miss a previously recalled item on the next trial, which may argue for increased forgetting and hence be in line with our findings of increased forgetting of degraded auditory information in healthy volunteers.

A wealth of experimental evidence has related working memory capacity to speech in noise performance (Akeroyd, 2008; Rönnberg et al., 2013; Rudner and Lunner, 2014). Further, individual differences in forgetting in elderly volunteers are strongly related to working memory capacity (Zimprich and Kurtz, 2013). We therefore investigated in a second analysis whether a high working memory capacity may attenuate the forgetting of degraded information and hypothesized that this may be especially relevant in elderly participants. Our results however did not support this hypothesis, since we found that only in young volunteers a high working memory capacity was related to forgetting of degraded information. In contrast, in old volunteers, verbal IQ but not working memory capacity predicted forgetting of degraded information. Even though that most of the prior research mentioned above indicated working memory capacity as a strong predictor of speech understanding in noise, there are several studies that report smaller effects of working memory and suggest other cognitive predictors. Noteworthy, one of those predictors was vocabulary size, which seems especially relevant in situations that require adaptation to or recognition of unfamiliar speech input (Erb et al., 2012; Janse and Adank, 2012; Banks et al., 2015; Carroll et al., 2016). Banks et al. (2015), who found that executive functions and vocabulary size predicted recognition of accented speech suggest that the effects of vocabulary size may be explained by an easier identification and better lexical access of degraded items as well as a better anticipation of upcomping information in subjects with greater vocabulary knowledge. We here show that different cognitive abilities contribute to forgetting of degraded information in young and old volunteers. While high working memory capacity may compensate for a potentially impoverished representation of degraded auditory information in young subjects, better lexical access may be the relevant factor in the elderly.

## Author Contributions

CMT designed the study, analyzed the data and wrote the manuscript. JÖ was involved in data acquisition and interpretation and revised the manuscript. WN was involved in parts of the design of the study, provided the vocoder routines and wrote this part of the methods. SP designed the study, was involved in data acquisition and interpretation and revised the manuscript. All authors approved the final version of the manuscript.

## Conflict of Interest Statement

The authors declare that the research was conducted in the absence of any commercial or financial relationships that could be construed as a potential conflict of interest.
